# Supplementary material for: Uncertainty and precaution in hunting wolves twice in a year
Source: PLoS One. 2022 Mar 16;17(3):e0259604. doi: 10.1371/journal.pone.0259604 (PMC8926205; doi:10.1371/journal.pone.0259604)
Supplement: S2 Table — Note that the formulae should follow the insertion of ‘ = ‘ to become active and then should be pasted into all cells within a sheet. Yellow sheets contain outputs of randomization, whereas white or gray sheets are user input fields. S2 Table presents the outputs of these algorithms in a single iteration used in the Results (1200 values) whereas results in Fig 4 represent three such iterations (3600 values). (PDF) [file pone.0259604.s003.pdf]

Deaths uniform distribution between 0.38-0.56

**RANDBETWEEN(38,56)÷100**									

Packs with pups normal centered 0.72 (range 0.55-0.89)

**(((RANDBETWEEN(1,17)+RANDBETWEEN(1,17))−2)÷100)+0.52**									

Litter size 4.8 (3-6) normal centered on 4.8

**TRUNC(((RANDBETWEEN(3,6)+RANDBETWEEN(3,6))÷2)+0.3)**									

Pup survival to 3-9 months normal, long right tail, mean 0.2 (0.05-0.72)

**(RANDBETWEEN(0,16)+RANDBETWEEN(0,16)+IF(RAND()<0.75,0,RANDBETWEEN(3,40)))÷100**									

Number of breeding packs 74-167 uniform random

**RANDBETWEEN(74,167)**									

N2021 traditional =695-751 uniform

RANDBETWEEN(695,751)									

N2021new =1195 (957-1573 normal) The 218 killed in Feb 2021 wolf-hunt are deducted in subsequent steps.

1075+(RANDBETWEEN(–496,756)+RANDBETWEEN(–496,756))÷2									

Death tolls user defined

**Any value from 0-600 could be entered here**									

Death tolls mean 300 normal distribution

**(RANDBETWEEN(0,300)+RANDBETWEEN(0,300))**								

Results (user defined death toll and paste in N2021)

**('N2021 traditional =695-751 uniform'::A1+('Number of breeding packs 74-167 uniform random'::A1×'Packs with pups normal centered 0.72 (range 0.55-0.89)'::A1×'Litter size 4.8 (3-6) normal centered on 4.8'::A1×'Pup survival to 3-9 months normal, long right tail, mean 0.2 (0.05-0.72)'::A1)×0.5)×(1−'Deaths uniform distribution between 0.38-0.56'::A1)−Death tolls user defined::A1**									

Results (traditional census method)

**('N2021 traditional =695-751 uniform'::A1+('Number of breeding packs 74-167 uniform random'::A1×'Packs with pups normal centered 0.72 (range 0.55-0.89)'::A1×'Litter size 4.8 (3-6) normal centered on 4.8'::A1×'Pup survival to 3-9 months normal, long right tail, mean 0.2 (0.05-0.72)'::A1)×0.5)×(1–'Deaths uniform distribution between 0.38-0.56'::A1)–Death tolls user defined::A1**									

Results (new census method)

**('N2021new =1195 (957-1573 normal) The 218 killed in Feb 2021 wolf-hunt are deducted in subsequent steps.'::A1−218+('Number of breeding packs 74-167 uniform random'::A1×'Packs with pups normal centered 0.72 (range 0.55-0.89)'::A1×'Litter size 4.8 (3-6) normal centered on 4.8'::A1×'Pup survival to 3-9 months normal, long right tail, mean 0.2 (0.05-0.72)'::A1)×0.5)×'Deaths uniform distribution between 0.38-0.56'::A1−Death tolls user defined::A1**

Pairs of values N2022 v H user-defined

**('N2021 traditional =695-751 uniform'::A1+('Number of breeding packs 74-167 uniform random'::A1×'Packs with pups normal centered 0.72 (range 0.55-0.89)'::A1×'Litter size 4.8 (3-6) normal centered on 4.8'::A1×'Pup survival to 3-9 months normal, long right tail, mean 0.2 (0.05-0.72)'::A1)×0.5)×(1−'Deaths uniform distribution between 0.38-0.56'::A1)−Death tolls user defined::A1**	**Any value from 0-600 could be entered here**
