# Supplementary material for: Uncertainty and precaution in hunting wolves twice in a year
Source: PLoS One. 2022 Mar 16;17(3):e0259604. doi: 10.1371/journal.pone.0259604 (PMC8926205; doi:10.1371/journal.pone.0259604)
Supplement: Attachment — Submitted filename: Review_response.pdf [file pone.0259604.s005.pdf]

PONE-D-21-32862

Dear Editor and reviewers
Thank you for the opportunity to revise and resubmit our paper. In boldface below, we provide point by point replies and remedies for the deficiencies identified by the editor and reviewers.

The reviewers raised only a few questions regarding this manuscript. They suggest a revision of figures and several supplementary explanations or rewording in the methodology.

Thank you. We made all the changes suggested with one exception (we did not add a new figure), which we explain. Revisions are specified below..

I also believe that the manuscript is of good quality; however, some parts require improvement. For example, please revise the references, as there are many errors (Tva v hill, Epstein Y. Killing wolves to save them? , Johnson RR, Curtis A...).

Apologies. The lead author's endnote software struggles with legal cases (e.g., TVA v Hill 1978). He will manually confirm each.

Furthermore, the Wikipedia article should be archived (https://archive.org/web/ and referenced with the link to the archived article.

We were unaware of this facility. Thank you for advisingWe created web archives for every URL except Wisconsin Public Radio and Official documents posted by the state government.

The introduction is followed by a chapter named Uncertainty. It is the intention, or should it be part of the intro?

We intended to use "Uncertainty" as a subheading to match the subheading named "Precaution". Here they are now correctly positioned and emphasized.

You stated in the letter to Plos One that the work will be controversial. I would like to introduce a short discussion on what is controversial in the paper and what you expect to trigger with this paper. This info is in the paper, but I think it deserves a more straightforth acknowledgment.

We interpreted the editor's suggestion as an invitation to add a section in the Discussion about the various controversies swirling around our subject and how we hope to address the controversies. We also saw the editor's recommendation that we separate out political discussion from the discussion of our results and methods. Therefore, we combined the discussion of controversy with any overt

discussion of politics in the former section. This required a major reorganization of the Discussion but we are pleased with the result. Thank you

An unmarked version of your revised paper without tracked changes. You should upload this as a separate file labeled ‘Manuscript'.

Done

If you would like to make changes to your financial disclosure, please include your updated statement in your cover letter.

We have updated and aligned our statements.

PLOS ONE requires that all conclusions in the manuscript must be supported by presented data (https://journals.plos.org/plosone/s/criteria-for-publication#loc-4). In this instance, please amend any statements, such as the last couple of sentences in the abstract, to ensure that they are not opinion based.

Done by deleting the penultimate sentence of the abstract. We also scrutinized the remainder of the paper for assertions of fact or opinions that do not flow from our research.

In addition, please ensure that the conclusions are based on the evidence shown here, staying away from political discussions and/or recommendations as much as possible.

Please see above. We do not understand the advice to avoid recommendations as this is standard in discussion sections. However, we have revised to avoid political recommendations and instead we make recommendations for scientific management.

Journal Requirements:

When submitting your revision, we need you to address these additional requirements.

1. Please ensure that your manuscript meets PLOS ONE's style requirements, including those for file naming. The PLOS ONE style templates can be found at https://journals.plos.org/plosone/s/file?id=wjVg/PLOSOne_formatting_sample_main_body.pdf and https://journals.plos.org/plosone/s/file?id=ba62/PLOSOne_formatting_sample_title_authors_affiliations.pdf

Done, thank you.

2. We note that the grant information you provided in the ‘Funding Information’ and ‘Financial Disclosure’ sections do not match. When you resubmit, please ensure that

you provide the correct grant numbers for the awards you received for your study in the 'Funding Information' section.

We apologize for any confusion. We stated, "AT was funded by DAAD: German Academic Exchange Service (Personal reference no. 91803225).
NXL was funded by the Natural Sciences and Engineering Research Council of Canada (PGSD3 - 545968 - 2020). Neither sure of funding was for this work specifically. This does not alter our adherence to PLOS ONE policies on sharing data and materials."

3. Thank you for stating the following in the Competing Interests section:

…[we deleted what we submitted initially, to make reading easier]…

Please confirm that this does not alter your adherence to all PLOS ONE policies on sharing data and materials, by including the following statement: "This does not alter our adherence to PLOS ONE policies on sharing data and materials." (as detailed online in our guide for authors http://journals.plos.org/plosone/s/competing-interests).

Done in the statement of potentially competing interests appended to our submission.

If there are restrictions on sharing of data and/or materials, please state these.

There are none.

Please note that we cannot proceed with consideration of your article until this information has been declared.

Please include your updated Competing Interests statement in your cover letter; we will change the online submission form on your behalf.

Done

4. Please amend the manuscript submission data (via Edit Submission) to include author Louchouarn, Naomi X.

Done

5. We note that you have referenced (ie. Bewick et al. [5]) which has currently not yet been accepted for publication. Please remove this from your References and amend this to state in the body of your manuscript: (ie "Bewick et al. [Unpublished]") as detailed online in our guide for authors
http://journals.plos.org/plosone/s/submission-guidelines#loc-reference-style

We can not see this citation anywhere within our manuscript. Citation [5] is Plater (2004). However, if you point out the appropriate location of the citation we are more than happy to make the change.

6. We note that Figure 1 in your submission contain [map/satellite] images which may be copyrighted. All PLOS content is published under the Creative Commons Attribution License (CC BY 4.0), which means that the manuscript, images, and Supporting Information files will be freely available online, and any third party is permitted to access, download, copy, distribute, and use these materials in any way, even commercially, with proper attribution. For these reasons, we cannot publish previously copyrighted maps or satellite images created using proprietary data, such as Google software (Google Maps, Street View, and Earth). For more information, see our copyright guidelines: http://journals.plos.org/plosone/s/licenses-and-copyright.

We require you to either (1) present written permission from the copyright holder to publish these figures specifically under the CC BY 4.0 license, or (2) remove the figures from your submission:

a. You may seek permission from the original copyright holder of Figure 1 to publish the content specifically under the CC BY 4.0 license.

We recommend that you contact the original copyright holder with the Content Permission Form (http://journals.plos.org/plosone/s/file?id=7c09/content-permission-form.pdf) and the following text:
"I request permission for the open-access journal PLOS ONE to publish XXX under the Creative Commons Attribution License (CCAL) CC BY 4.0 (http://creativecommons.org/licenses/by/4.0/). Please be aware that this license allows unrestricted use and distribution, even commercially, by third parties. Please reply and provide explicit written permission to publish XXX under a CC BY license and complete the attached form."

Please upload the completed Content Permission Form or other proof of granted permissions as an "Other" file with your submission.

In the figure caption of the copyrighted figure, please include the following text:
"Reprinted from [ref] under a CC BY license, with permission from [name of publisher], original copyright [original copyright year]."

b. If you are unable to obtain permission from the original copyright holder to publish these figures under the CC BY 4.0 license or if the copyright holder's requirements are incompatible with the CC BY 4.0 license, please either i) remove the figure or ii) supply a replacement figure that complies with the CC BY 4.0 license. Please check copyright information on all replacement figures and update the figure caption with source information. If applicable, please specify in the figure caption text when a figure is similar but not identical to the original image and is therefore for illustrative purposes only.
The following resources for replacing copyrighted map figures may be helpful:

USGS National Map Viewer (public domain): http://viewer.nationalmap.gov/viewer/
The Gateway to Astronaut Photography of Earth (public domain): http://eol.jsc.nasa.gov/sseop/clickmap/
Maps at the CIA (public domain): https://www.cia.gov/library/publications/the-world-factbook/index.html and https://www.cia.gov/library/publications/cia-maps-publications/index.html
NASA Earth Observatory (public domain): http://earthobservatory.nasa.gov/
Landsat: http://landsat.visibleearth.nasa.gov/
USGS EROS (Earth Resources Observatory and Science (EROS) Center) (public domain): http://eros.usgs.gov/#
Natural Earth (public domain): http://www.naturalearthdata.com/

The Wisconsin county map seen in Figure 1 is a shapefile provided by the Wisconsin Department of Natural Resources Open Data Portal. It therefore is not, under copyright. We have added the source link to the figure caption.

Further, NXL created the wolf pack map by geo-referencing reported wolf pack locations in the state. Therefore, these data layers were created by us, and we do not replicate the Wisconsin DNR's maps (though we do cite them in our figure caption). Therefore, we have copyright.

7. Please review your reference list to ensure that it is complete and correct. If you have cited papers that have been retracted, please include the rationale for doing so in the manuscript text, or remove these references and replace them with relevant current references. Any changes to the reference list should be mentioned in the rebuttal letter that accompanies your revised manuscript. If you need to cite a retracted article, indicate the article's retracted status in the References list and also include a citation and full reference for the retraction notice.

NA

[Note: HTML markup is below. Please do not edit.]

Reviewers' comments:

Reviewer's Responses to Questions

Comments to the Author

1. Is the manuscript technically sound, and do the data support the conclusions?

Reviewer #1: Yes

Reviewer #2: Yes

---

2. Has the statistical analysis been performed appropriately and rigorously?

Reviewer #1: Yes

Reviewer #2: Yes

---

3. Have the authors made all data underlying the findings in their manuscript fully available?

Reviewer #1: Yes

Reviewer #2: Yes

---

4. Is the manuscript presented in an intelligible fashion and written in standard English?

Reviewer #1: Yes

Reviewer #2: Yes

---

5. Review Comments to the Author

Reviewer #1: General comments on the manuscript
It was a pleasure reading this manuscript. The manuscript is constructed to allow science to have a practical input therefore the conclusions are supported by the data, while ethical and logical arguments are supporting the study objectives and the discussion. The fact that the manuscript is actually describing the uncertainties in relation to precautions to explain how one can get from basic data to conclusions plays a methodological and an informal role. Since one of the manuscript objective is to inform not only scientist but also wildlife managers and policy makers on social sensitive topics like hunting quotas keeping the statistical tools at a basic level is highly relevant for the manuscript and it is a good editorial strategy.

General suggestions for the authors

I believe the title is not reflecting at all the second goal of the manuscript related to the potential of the method being replicated for other species. I would suggest a new title to

incorporate the recommendations for scientist maybe something like: "Uncertainty and precaution in hunting management should build a bridge between science, politics and decision making."

We recognize the wisdom the suggestion but choose a different remedy because the word "wolves" in the title raises the likelihood of attention from our primary audiences. Also, short titles seem to us to be read more carefully and cited more often (evidence from the authors' own works). Instead of changing the title, we added a keyword "wildlife populations", a sentence to the abstract, and a sentence to the introduction that make the reviewer's point more effectively we feel.

I believe your work is a perfect example to explain how the concept of "political population" (Darimont et al. 2018) works in practice and also how cultural differences lead to different perspective on the same object, in this case how the Red cliff government has a different perspective. I would recommend to explore more this two ideas in the Introduction and also in the Discussion. In the end Uncertainty is also a result of social and professional culture. From my understanding the concept of political population is a powerful concept and used wisely can support strategically the influence of science on policies.

We added a summary of the concept of political populations to the Discussion in the paragraph on controversy. Also in the Introduction, as recommended by the reviewer, we define a political population and state the following "Relatedly, concerns have been expressed by scientists and managers about 'political populations' defined as wildlife whose population parameters are set by political pressures despite being biologically unrealistic {Darimont, 2018 #2344}. Scientific work that can bridge between biological (or social scientific) observations on the one hand, and management or policy-making on the other hand, may help to minimize undue political pressure." And "One manifestation of such political pressures is the tendency for agencies to report unrealistic biological parameters that appear to the uninformed to support claims that killing is 'sustainable' or 'safe'. Such "political populations" {Darimont, 2018 #2344} seem designed to satisfy political demands by inflating population parameters of the carnivores targeted for killing."

On relation to this proposal I believe it will be more clear if you would explain the three thresholds (<2, <251, <350) separately and not in the Uncertainty section in the Introduction since they are social and political justified.

Thank you for this excellent suggestion. We moved the threshold definitions up to the section on precaution. This makes more sense precisely for the reasons articulated by the reviewer and also serve to clarify why we are not interposing our own values when we use legal thresholds as the criteria for precautionary approaches.

I would suggest also to find a way to explain graphically all the values and ranges for your estimation of potential values for R an M to help the readers to follow the algorithm easily.

For R, there is Figure 2 and all other parameters are simple mean and 95% CI values so we don't think another figure would be useful.

For M, we feel Table 1 does this adequately. To help the reader we added a sentence at the end of the methods on deaths, stating "We used a uniform distribution analogous to Figure 2 for D." Given that we provided the values and rationale from the peer-reviewed literature for the range of values along that uniform distribution, we opted not to present it graphically again.

Note we also added a sentence to the caption for Figure 2 noting the above analogous procedure for deaths.

Specific comments on the manuscript
line 72 - Without speculating I would mention main causes of dead for 98-105 wolves.

Done

line 238 - why did you assume only 218 wolves and not use also the other 99 overshot? please explain.

The simple answer is that the state denied or ignored the additional deaths (98-105) that we modeled and published. Because we provide two scenarios (the state's and ours) we used their assumptions for the former.

line 295 - the value of harvested wolves is 218 and the number of packs that could have breed is also 218 and this creates a little bit of confusion in the manuscript. Please find a way to eliminate this confusion.

Good catch—as the reviewer surmised this is a coincidence. Given the number of packs was not a whole number, we presented it with its decimals (218.05) in the 3 sentences in which it appears and Figure 2. We hope this will disambiguate the coincidentally similar values.

296 - not sure if the 218 packs failed to bread or were potential breeding. In the first scenario and compared with the line 292 statement and following results seems that the second context is the right one. Can you please check this statement?

Clarified that 218.05 is a maximum (implausible) number of packs that bred. We confirmed the clarity of text on this issue.

line 503 - please check the number of the figure.

Another god catch, thank you. corrected.

line626 - maybe it is driven

We changed our wording thanks

Conclusion
I believe the manuscript is suitable for publishing in PLOSone, fulfilling the journal criteria and since it covers methodological aspects that can be replicated and it is promoting the improvement of research quality it is highly relevant for several professional groups. Really hope to see this beautiful mixed of math science, ethics and anthropology published and used by scientist to promote better policies.

Thank you, so do we!

Reviewer #2: The article tackles a highly important and critical aspect of wolf (and wildlife in general) harvesting decision-making processes. Hunting animals with a relatively slow life history and a relatively high ecological function (such as large carnivores, which could trigger ecosystem-level changes) necessitates caution and thoughtful consideration. Because these processes are sometimes driven by political interests rather than scientific evidence, a critical and robust assessment of the effects of harvesting is vital for species persistence. In this context, the article makes an interesting contribution by providing a qualitative and quantitative analysis of the effect of harvesting Wisconsin wolves using both outdated quotas and adding an additional hunting season.

Despite the lack of reliable demographic data, the authors put up a significant amount of work in parameterizing the model. The authors performed a remarkable effort in finding the best possible parameters from different sources (i.e. grey literature) and compiling them in a meaningful way. Both immigration and emigration data, as well as non-linear relationship shapes, are not taken into account (yet critical in modelling population trends), but the authors present a good explanation for this methodological decision. The use of an uninformative uniform distribution helped reduce potential biases and, importantly, the authors used bootstrapping to account for parameter uncertainty, which is essential for the final interpretation of the results.

Thank you.

The manuscript is overall well organized and easy to read, it is well structured logically and linguistically, while the arguments are supported by appropriate literature. There are no major comments on the methodology used as is fully in line with the scope of the manuscript. As indicated by the authors, the work will most certainly be divisive due to its nature and message (i.e. zero death toll). While the statements are likely to be interpreted as political (particularly “Politics of precautions and the role of science") , I believe the authors gave a reasonable justification for their standpoint.

Thanks

The following comments are offered with the purpose of improving the manuscript:

Figure 1 - Add scale bar and north reference. Add A) and B) for consistency with the figure caption. The use of grey for both “affected wolf pack” and administrative borders (county?) is misleading. Either refer to the grey circle in the legend for “affected wolf pack”, or change color. Could the author report the average wolf territory size used for the spatial overlay and geo-rectification?

Done

Figure 2 - The figure caption seems to be missing. The space interval between values in the x-axis is completely arbitrary, and thus incorrect. Regardless of the figure's theoretical basis, manipulating the intervals artificially is to be avoided.

The caption appears on the page following so we apologize if this caused confusion. We agree the spacing was imperfect and have corrected it.

Figure 4 - y-axis values are missing.

Done

---

6. PLOS authors have the option to publish the peer review history of their article (what does this mean?<https://journals.plos.org/plosone/s/editorial-and-peer-review-process#loc-peer-review-history>). If published, this will include your full peer review and any attached files.

Yes please

While revising your submission, please upload your figure files to the Preflight Analysis and Conversion Engine (PACE) digital diagnostic tool, https://pacev2.apexcovantage.com/. PACE helps ensure that figures meet PLOS requirements. To use PACE, you must first register as a user. Registration is free. Then, login and navigate to the UPLOAD tab, where you will find detailed instructions on how to use the tool. If you encounter any issues or have any questions when using PACE, please email PLOS at figures@plos.org<mailto:figures@plos.org>. Please note that Supporting Information files do not need this step.

Done
