# Supplementary material for: Uncertainty and precaution in hunting wolves twice in a year
Source: PLoS One. 2022 Mar 16;17(3):e0259604. doi: 10.1371/journal.pone.0259604 (PMC8926205; doi:10.1371/journal.pone.0259604)
Supplement: Attachment — Submitted filename: Response to Reviewers.docx [file pone.0259604.s004.docx]

Dear editors of PLoS One,

Please consider our research article entitled, “Uncertainty and precaution in hunting gray wolves twice in one year”. The following questions from PLoS Biology might be useful in your evaluation of whether to send or ums. For review.

- What is the scientific question you are addressing?
- **We addressed how scientists should account transparently for uncertainty so that decision-makers can apply precautionary approaches to public policy. We address this global question with a case study of the Wisconsin gray wolf population recently delisted from the U.S. Endangered Species Act (ESA).**
- What is the key finding that answers this question?
- **Scientific uncertainty often hinders precautionary approaches. Yet policy-makers are often forced to decide anyway. If scientists turn away from public policy debates characterized by wide gaps in data or great uncertainty, then decision-makers may decide based on opinion, anecdote, or political pressures. We aim to bolster scientists’ confidence in their ability to grapple with uncertainty in a way useful to public policy.**
- **We use a case study of a recently delisted endangered species subjected to hunting to illustrate the use of legal thresholds to define the probabilities that policy will result in undesirable effects. Societal value judgments have produced legal thresholds that decide what is precautionary and what is not, relieving scientists of the appearance of making personal value judgments when evaluating policy effects.**
- **We address the probabilities of crossing three legal thresholds with simple models and Bayesian concepts to account for uncertainty. We demonstrate constructive approaches to using an interdisciplinary mix of qualitative and quantitative information to reduce uncertainty to manageable levels with uninformative, uniformly distributed priors. We explain our priors in terms that the public and policy-makers can grasp and present our simple models in ways the untrained can use to explore their own scenarios. In sum, we translate the technical process of modeling with uncertainty in a way that can transfer to other fields.**
- **We address these global questions with a case study of the Wisconsin gray wolf population recently delisted from the ESA. Our approach is rather novel in our field of predator ecology, which has been more concerned with sustainability of hunting. We show that state policies have a high probability of undesirable outcomes judged by legal thresholds. We conclude that holding a second wolf-hunt in 2021 with any death toll over 16 wolves has more chance than not of crossing an undesirable legal threshold. We show how the state government’s quotas would risk undesirable thresholds including extinction in the state.**
- **Although our work will be controversial (everything seems controversial about wolves), we expect to transform the debate over whether carnivore-hunts are sustainable to debates about the risk of crossing undesirable legal thresholds. Probabilities with estimates of uncertainty are superior to claims that a hunt is sustainable or unsustainable because the public and policy-makers can glimpse risk in probabilistic terms and debate the advisability of crossing legal thresholds. Moreover, we take pains to explain the influence of uncertainty on our models in a manner that can help the public and policy-makers to implement precautionary approaches.**
- What is the nature of the evidence you provide in support of your conclusion?
- **We model population change, birth and mortality rates, and impose wolf-hunting scenarios on the outputs to quantify the probability that the state wolf population crosses legal thresholds including state listing as endangered, extinction in the state, and dropping below the state population goal.**
- What are the three most recently published articles that are relevant to this question?

**Creel, S., M. Becker, D. Christianson, E. Dröge, N. Hammerschlag, M.W. Hayward, K.U. Karanth, A.J. Loveridge, D. Macdonald, W. Matandiko, J. M’soka, D. Murray, E. Rosenblatt, and P. Schuette, Questionable policy for large carnivore hunting. Science, 2015. 350(6267):1473-1475.**

**Li, Y.-W., J. Roman, D.S. Wilcove, T. Male, and H. Doremus, Species protection will take more than rule reversal. Science, 2020. 370(6517): 665-666.**

**Treves, A., F.J. Santiago-Ávila, and K. Putrevu, Quantifying the effects of delisting wolves after the first state began lethal management. PeerJ, 2021. 9: p. e11666. 10.7717/peerj.11666.**

- What significance do your results have for the field?
- **For carnivore science, we address a lingering debate about sustainability that has become arid in the absence of clear legal thresholds or has become arid because of competing results by different research teams.**
- **We recommend that scientists practice analysis and communication that improves their ability to explain what the uncertainty means for policy and the public. We suggest a hypothesis that the more uninformative the prior data, the more scenarios one should present and the more transparent the assumptions about inputs should be made — if the goal is science-informed policy.**
- What significance do your results have for the broader community (of biologists and/or the public)?
- **Scientists may turn away from public policy debates when data are not available regardless of the importance of the debates.**
- **The benefit of our approach is that untrained users can manipulate our models for themselves, because we forced ourselves to use the simplest terms and algorithms appropriate to the problem.**
- What other novel findings do you present?
- **We present the most recent estimate for wolf mortality in Wisconsin since 2013. Our estimate is important beyond Wisconsin and beyond wolves because the field has been debating the magnitude of the differences in mortality between marked (e.g., collared) animals and unmarked ones. We add to this debate by providing a case in which estimates of mortality for marked wolves were as low as 23% but estimates for unmarked wolves range from 38-56%. Thus our findings join results from Scandinavian wolverines and Alaskan wolves that suggest the major cause of carnivore mortality (humans) is somehow reduced by marking wild animals. The implications for investigators worldwide are arresting because we may one day find out that research was promoting conservation but biasing our science.**
- Is there additional information that we should take into account?
- **Wolves are in the news particularly in Wisconsin this year. After the first wolf-hunt in February 2021 I counted 175 news reports. The decision to hunt wolves again in August 2021 triggered 550 news articles around the world. We expect this article to draw attention to an important issue in the nexus of policy and science.**

Opposed reviewers

There are three federal lawsuits relating to wolves at resent. Therefore, I oppose reviewers currently employed or employed in the past by the federal wildlife agencies including U.S. Department of Agriculture and U.S. Geological Survey if the authors have conducted wildlife research or Wisconsin. I oppose employees of the State of Wisconsin Department of Natural Resources. I oppose current or former staff of the Great Lakes Indian Fish & Wildlife Commission.

There is also a state lawsuit on which I intend to submit an expert declaration (see competing interest declaration. I oppose parties to that litigation.
